# A153 PYCRL LACTYLATION AS A POTENTIAL REGULATOR OF CANCER STEM CELL METABOLISM IN ESOPHAGEAL SQUAMOUS CELL CARCINOMA (ESCC)

**DOI:** 10.1093/jcag/gwac036.153

**Published:** 2023-03-07

**Authors:** J Douchin, L G Nogueira de Almeida, A Gonneaud, F -M Boisvert, A Dufour, V Giroux

**Affiliations:** 1 Department of Immunology and Cell Biology, Faculty of Medicine and Health Sciences, Université de Sherbrooke; 2 Centre de Recherche du CHUS, Sherbrooke; 3 Departments of Physiology & Pharmacology and Biochemistry & Molecular Biology, University of Calgary, Calgary; 4 Departments of Physiology & Pharmacology and Biochemistry & Molecular Biology, University of Calgary, Sherbrooke, Canada

## Abstract

**Background:**

Patients with esophageal malignancy have a 5-year survival rate of only 14% in Canada. This high mortality rate is due to three factors: late diagnosis, difficulty in surgically removing the tumor because of its localization, and treatment resistance. Resistance can be developed after prolonged exposure to anti-cancer drugs and/or radiation. Indeed 30% of patients will not respond to treatment or will relapse. Resistance has been mainly ascribed to the presence of cancer stem cells (CSCs) inside the tumor. However, no treatment specifically directed against CSCs is available to patients.

**Purpose:**

Thus, targeting CSCs is a promising strategy to improve the survival of patients with esophageal squamous cell carcinoma (ESCC), the most common type of esophageal cancer worldwide. Therefore, a better understanding of the molecular mechanisms occurring during long-term exposure to cancer treatments is imperative.

**Method:**

Herein, we developed an unbiased approach to identify new players in chemotherapy and radiotherapy resistance development in ESCC. We established radio- (R), chemo- (C), and radiochemo-resistant (RC) human ESCC cell lines using prolonged exposure to radiation and/or chemotherapeutic agent 5-FU, respectively.

**Result(s):**

The enrichment in CSCs in all treated cell lines was demonstrated by an increase of ALDH1^high^ cells and CD24^high^/CD44^high^ cells in flow cytometry. We then used a proteomic approach to identify new players in treatment resistance. Interestingly, pathway analysis pointed out to alterations in energy metabolism as well as amino acid metabolism. Seahorse assays showed that resistant cell lines have a lower respiration rate than control cells, while glycolysis remains unchanged. To further characterize these metabolic changes, we performed an unbiased metabolomic study and confirmed a decrease in amino acid levels such as proline, in resistant cell lines. Recently, metabolic regulation has been linked to a new post-translational modification, lactylation. Proteomic data were re-analyzed looking for lactylated protein and found, amongst others, PYCRL, an enzyme implicated in proline biosynthesis, as one of the most differentially lactylated proteins in treated cell lines compared to control. PYCRL lactylation was confirmed using immuno-fluorescence colocalization and immuno-precipitation. Lastly, using AlphaFold, preliminary results point toward the importance of PYCRL lactylation impairing PYCRL homomultimerization.

**Conclusion(s):**

To conclude, our results suggest an important role of proline metabolism following long-term treatment in ESCC. This study is a first step toward the identification of new targets to fight treatment resistance in ESCC patients.

**Please acknowledge all funding agencies by checking the applicable boxes below:**

CAG, CIHR, Other

**Please indicate your source of funding;:**

FRQ

**Disclosure of Interest:**

None Declared

